# Supplementary material for: Dietary probiotic and synbiotic supplementation starting from maternal gestation improves muscular lipid metabolism in offspring piglets by reshaping colonic microbiota and metabolites
Source: mSystems. 2024 May 20;9(6):e00048-24. doi: 10.1128/msystems.00048-24 (PMC11237649; doi:10.1128/msystems.00048-24)
Supplement: Supplemental material — Supplemental figures and tables. [file msystems.00048-24-s0001.docx]

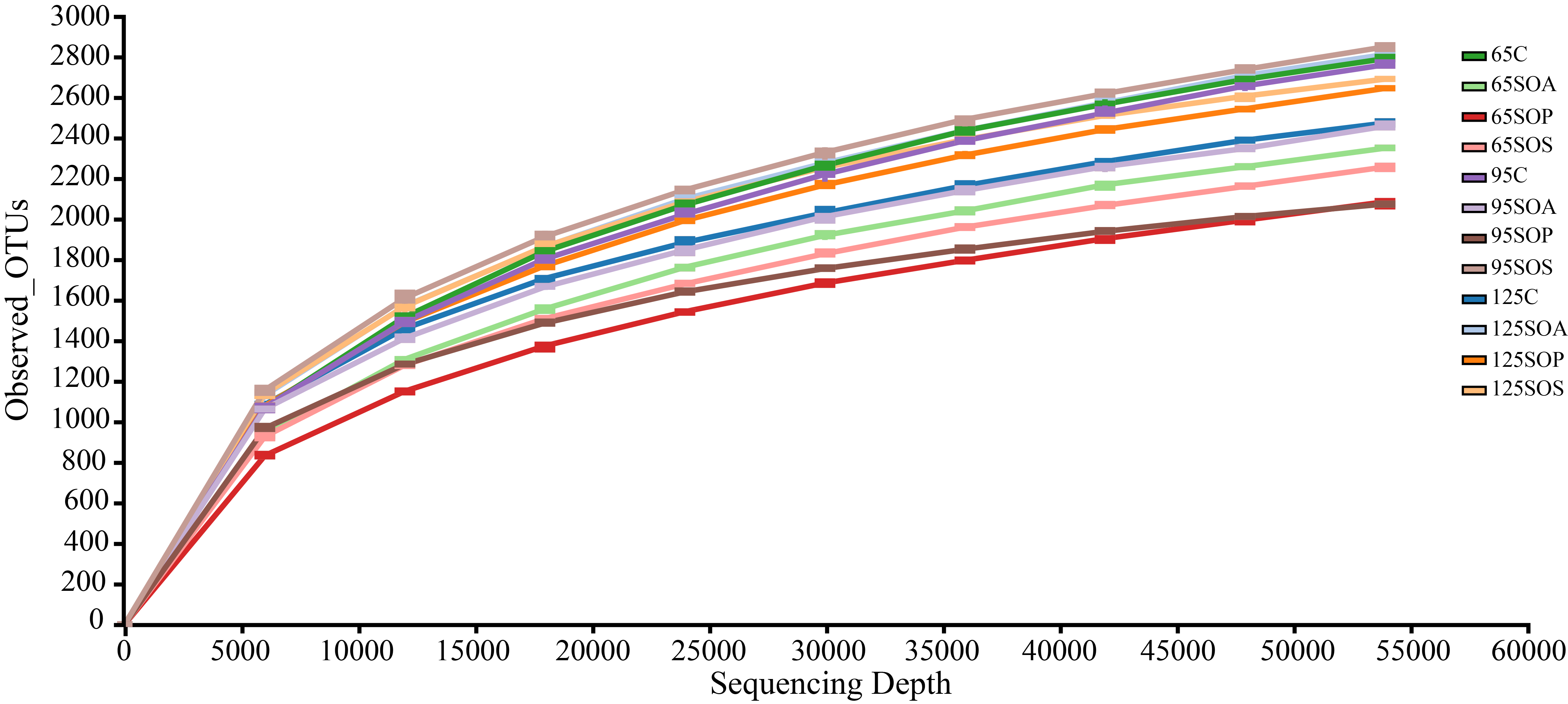


**Fig. S1** Rarefaction curves comparing the number of sequences with the number of phylotypes found in the 16S rRNA gene libraries from the microbiota in the colonic contents of offspring piglets. 65, 95, and 125 represent different day-old. C, control group; SOA, antibiotic supplementation in sow-offspring diets; SOP, probiotics supplementation in sow-offspring diets; SOS, synbiotics supplementation in sow-offspring diets. The replicates per group at 65 day-old were 8. The replicates of the C, SOA, SOP, and SOS groups at 95 day-old were 8, 8, 8, and 7, respectively. The replicates of the C, SOA, SOP, and SOS groups at 125 day-old were 8, 5, 6, and 6, respectively.


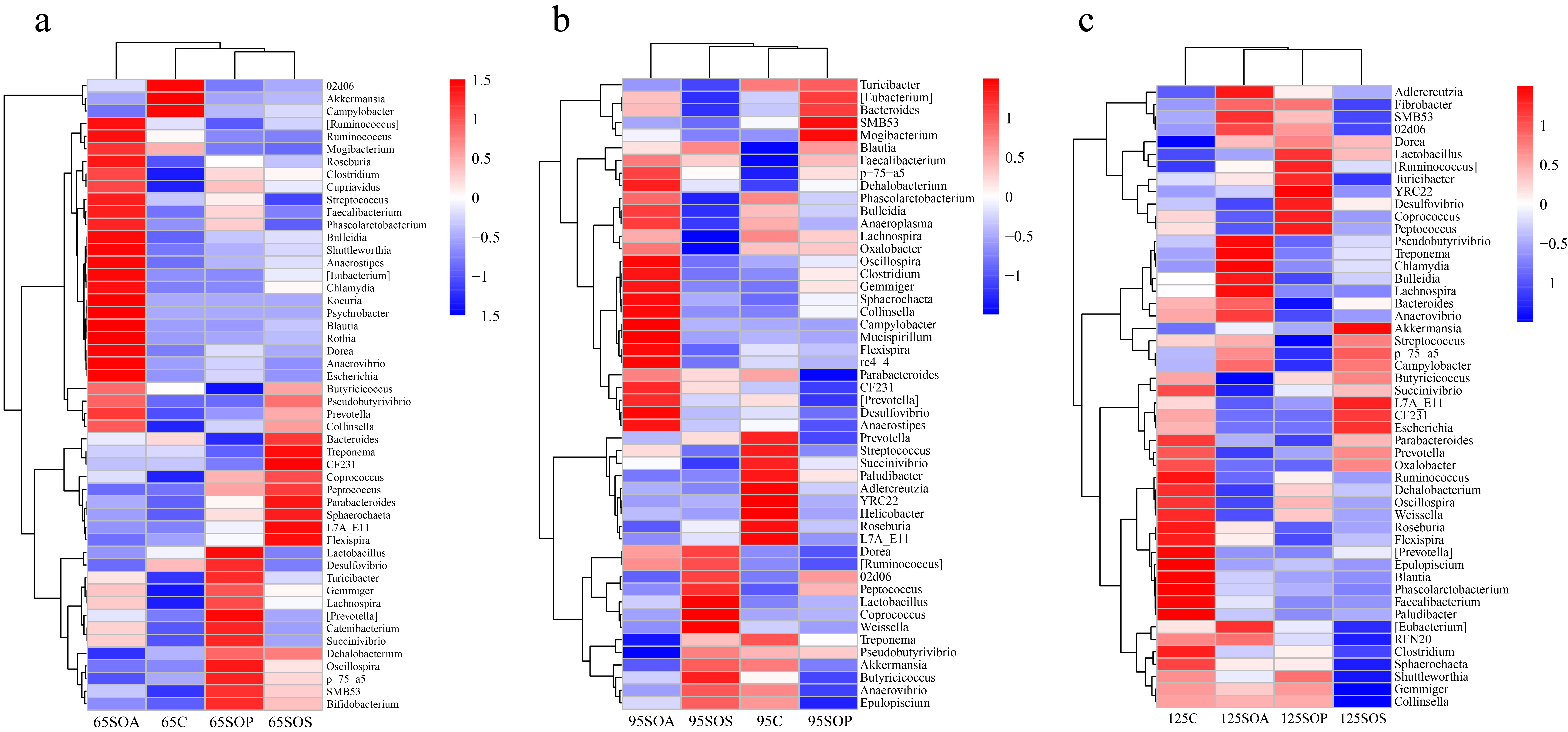


**Fig. S2** Hierarchical clustering and heatmap of colonic bacterial taxonomic composition at the genus level (the top 50 genera) of offspring piglets at 65 (a), 95 (b), and 125 (c) day-old. C, control group; SOA, antibiotic supplementation in sow-offspring diets; SOP, probiotics supplementation in sow-offspring diets; SOS, synbiotics supplementation in sow-offspring diets. The replicates per group at 65 day-old were 8. The replicates of the C, SOA, SOP, and SOS groups at 95 day-old were 8, 8, 8, and 7, respectively. The replicates of the C, SOA, SOP, and SOS groups at 125 day-old were 8, 5, 6, and 6, respectively.


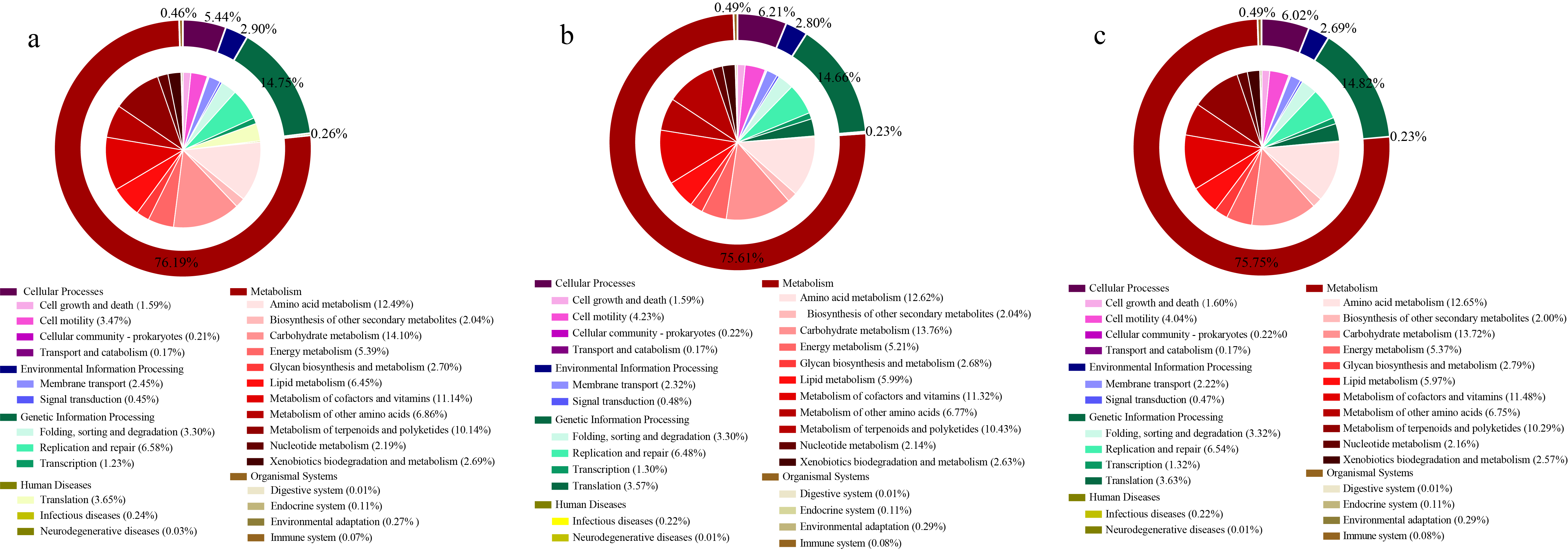


**Fig. S3** Predictive metagenomics showing the composition of function pathways at 65 (a), 95 (b), and 125 (c) day-old using the PICRUSt2 analysis at level 1 and level 2. Different color represents different metabolic pathway. The outer ring around the pie chart depicts the relative abundance of different pathway at level 1 and the inner circle show the relative abundance of different pathway at level 2.





**Fig. S4.** Principal component analysis (PCA) plot based on the colonic metabolites. PCA plot of the colonic metabolites in positive (a−c) and negative (d−f) models at 65, 95, and 125 day-old among treatment groups. C, control group; SOA, antibiotic supplementation in sow-offspring diets; SOP, probiotics supplementation in sow-offspring diets; SOS, synbiotics supplementation in sow-offspring diets. The replicates per group at 65 day-old were 8. The replicates of the C, SOA, SOP, and SOS groups at 95 day-old were 8, 8, 8, and 7, respectively. The replicates of the C, SOA, SOP, and SOS groups at 125 day-old were 8, 5, 6, and 6, respectively.


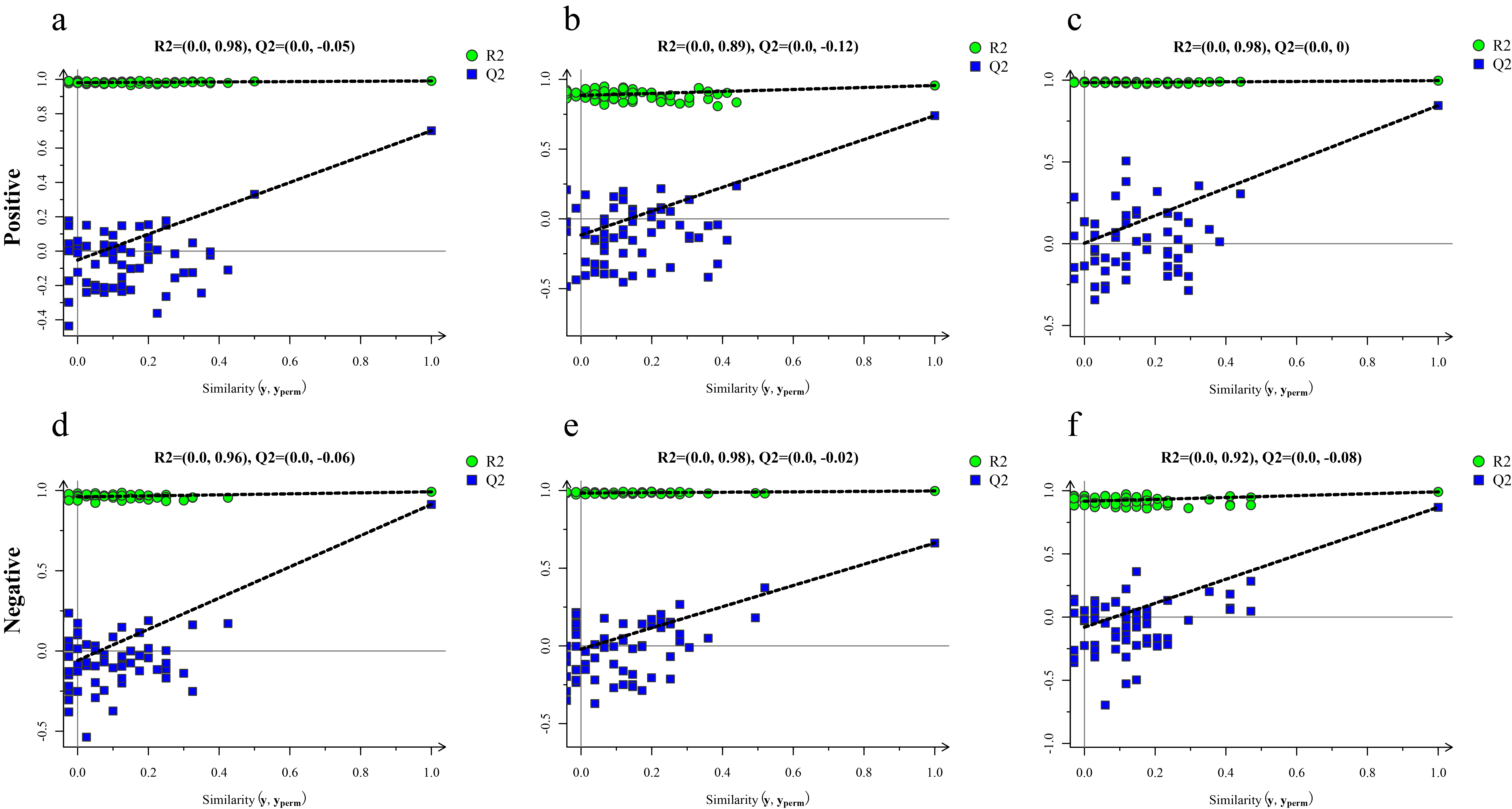


**Fig. S5.** The permutations plot of orthogonal partial least squares discriminant analysis (OPLS-DA). Permutations plot in positive (a−c) and negative (d−f) at 65, 95, and 125 day-old.


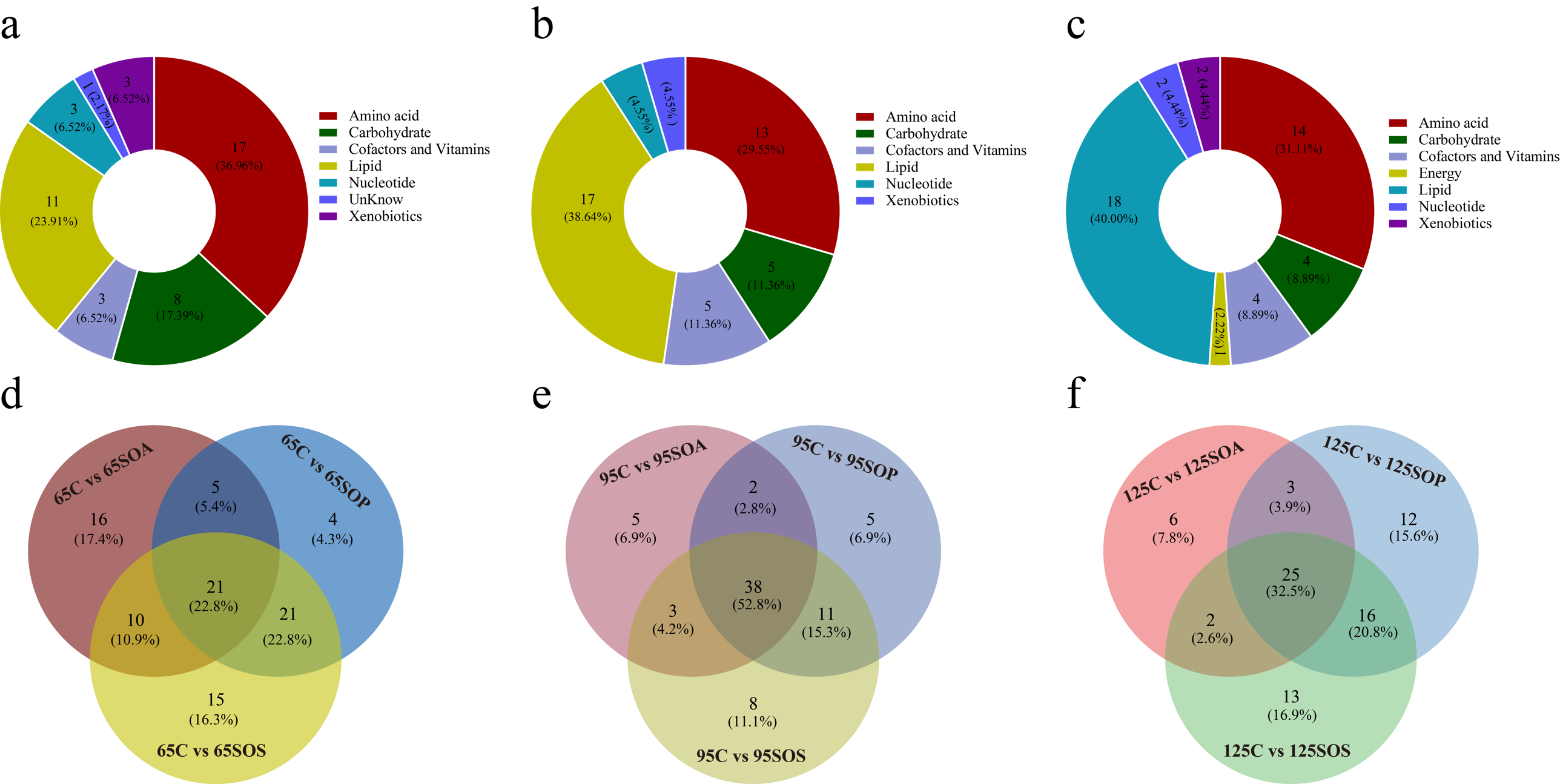


**Fig. S6** Differential metabolite analysis. Pie chart of differential metabolite classification at 65, 95, and 125 day-old (a−c). Venn diagram of differential metabolites for pairwise comparison (d−f). C, control group; SOA, antibiotic supplementation in sow-offspring diets; SOP, probiotics supplementation in sow-offspring diets; SOS, synbiotics supplementation in sow-offspring diets. The replicates per group at 65 day-old were 8. The replicates of the C, SOA, SOP, and SOS groups at 95 day-old were 8, 8, 8, and 7, respectively. The replicates of the C, SOA, SOP, and SOS groups at 125 day-old were 8, 5, 6, and 6, respectively.


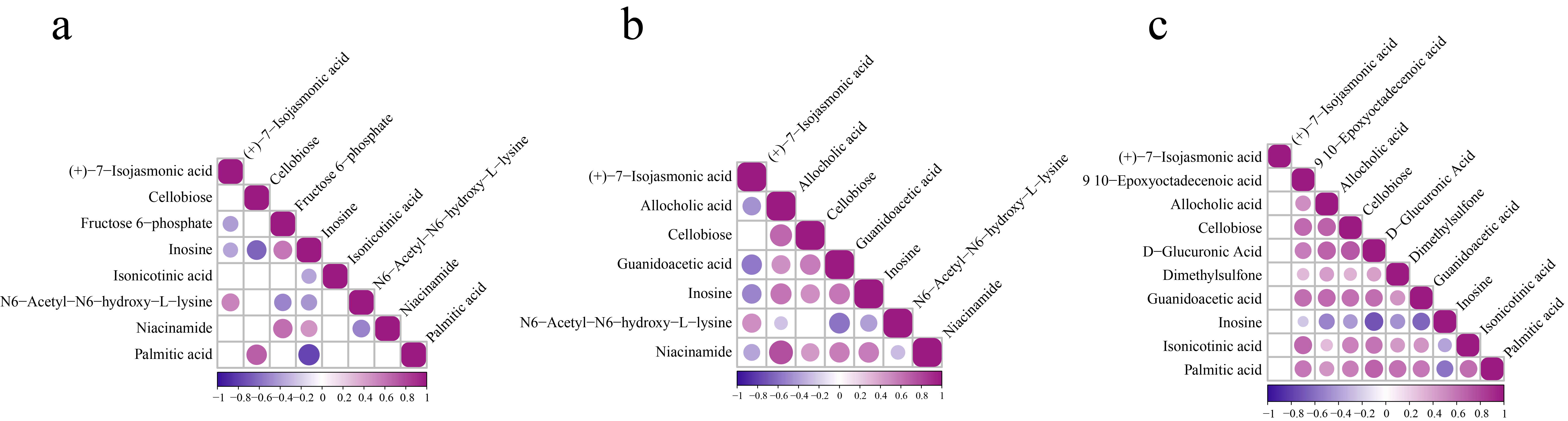


**Fig. S7** Correlation analysis of differential metabolite at 65 (a), 95 (b), and 125 (c) day-old. The maroon represents a significant positive correlation, and the blue represents a significant negative correlation.

**Table S1** Composition and nutrient levels of basal diets for sows (air-dry basis; %).

| **Items** | **Pregnant diet** | **Lactating diet** |
| --- | --- | --- |
| Ingredients |  |  |
| Corn | 37.50 | 66.00 |
| Soybean meal | 9.50 | 25.00 |
| Wheat bran | 14.00 | 5.00 |
| Barley | 25.00 |  |
| Soybean hull | 10.00 |  |
| Pregnant premix^1)^ | 4.00 |  |
| Lactating premix^2)^ |  | 4.00 |
| Total | 100.00 | 100.00 |
| Nutrient levels^3)^ |  |  |
| Digestible energy, MJ/Kg | 12.55 | 13.87 |
| Crude protein | 12.82 | 16.30 |
| Crude fiber | 4.56 | 2.87 |
| SID^4)^ Lys | 0.48 | 0.75 |
| SID Met+ Cys | 0.43 | 0.51 |
| SID Thr | 0.37 | 0.53 |
| SID Trp | 0.13 | 0.17 |
| Calcium | 0.62 | 0.65 |
| Phosphorus | 0.47 | 0.50 |

^1)^ Pregnant premix provided the following per kg of diet: CaHPO_4_⋅2H_2_O 10 g, NaCl 4 g, CuSO_4_⋅5H_2_O 80 mg, FeSO_4_ ⋅H_2_O 360 mg, ZnSO_4_⋅H_2_O 240 mg, MnSO_4_⋅H_2_O 100 mg, MgSO_4_⋅7H_2_O 1 g, 1% ICl 50 mg, 1% Na_2_SeO_3_ 36 mg, 1% CoCl_2_ 16 mg, NaHCO_3_ 1.4 g, VA 10,000 IU, VD_3_ 1800 IU, VE 20 mg, VK_3_ 2.4 mg, VB_1_ 1.6 mg, VB_2_ 6 mg, VB_6_ 1.6 mg, VB_12_ 0.024 mg, folic acid 1.2 mg, nicotinamide 20 mg, pantothenic acid 12 mg, biotin 0.12 mg, ferrous glycinate 100 mg, choline chloride 1g, phytase 200 mg, fruity 80 mg, and limestone 12 g.

^2)^ Lactating premix provided the following per kg of the diet: CaHPO_4_⋅2H_2_O 10 g, NaCl 4 g, CuSO_4_⋅5H_2_O 80 mg, FeSO_4_ ⋅H_2_O 360 mg, ZnSO_4_⋅H_2_O 240 mg, MnSO_4_⋅H_2_O 100 mg, 1% ICl 50 mg, 1% Na_2_SeO_3_ 36 mg, 1% CoCl_2_ 16 mg, NaHCO_3_ 1.4 g, VA 10,000 IU, VD_3_ 1,800 IU, VE 20 mg, VK_3_ 2.4 mg, VB_1_ 1.6 mg, VB_2_ 6 mg, VB_6_ 1.6 mg, VB_12_ 0.024 mg, folic acid 1.2 mg, nicotinamide 20 mg, pantothenic acid 12 mg, biotin 0.12 mg, lysine 1.5 g, ferrous glycinate 100 mg, choline chloride 1g, phytase 200 mg, fruity 80 mg, and limestone 12 g.

^3)^ Nutrient levels were calculated values.

^4)^ SID: standard ileum digestible.

**Table S2** Composition and nutrient levels of basal diets for weaned Bama mini-pigs (air-dry basis; %).

| **Items** | **Prophase diet**  **(35−95 day-old)** | | **Anaphase diet**  **(96−125 day-old)** |
| --- | --- | --- | --- |
| Ingredients | | | |
| Corn | | 54.92 | 58.00 |
| Soybean meal | | 22.00 | 18.35 |
| Wheat bran | | 10.13 | 11.35 |
| Rice bran | | 8.95 | 8.30 |
| Premix^1)^ | | 4.00 | 4.00 |
| Total | | 100.00 | 100.00 |
| Nutrient levels^2)^ | | | |
| Digestible energy, MJ/kg | | 13.50 | 13.42 |
| Crude protein | | 16.13 | 14.90 |
| Calcium | | 0.45 | 0.44 |
| Total Phosphorus | | 0.49 | 0.49 |
| Lys | | 1.40 | 1.30 |
| Met + Cys | | 0.69 | 0.66 |
| Thr | | 0.78 | 0.74 |

^1)^ Premix provided the following per kilogram of diets: enzyme preparation (including phytase, protease, and lipase) 1.2 g, VA 26,000 IU, VD_3_ 10,000 IU, VE 70 IU, VK_3_ 10 mg, VB_1_ 10 mg, VB_2_ 25 mg, VB_6_ 10 mg, VB_12_ 0.075 mg, biotin 0.4 mg, folic acid 5 mg, nicotinamide 100 mg, pantothenic 50 mg, choline 1600 mg, flavoring agent 500 mg, edulcorant 300 mg, acidulating agent 5 g, Cu (as CuSO_4_·5H_2_O) 23 mg, Mn (as MnSO_4_·H_2_O) 97 mg, Zn (as ZnSO_4_·H_2_O) 218 mg, Fe (as FeSO_4_·H_2_O) 165 mg, I (as Ca(IO_3_)_2_) 0.3 mg, Se (as Na_2_SeO_3_) 0.3 mg, Co (as CoSO_4_·H_2_O) 0.4 mg, glucose 2.1 g, antioxidants 0.4 g, anti-mildew agent 1 g, Ca (as CaHPO_4_ and CaCO_3_) 3.42 g, and P (as CaHPO_4_) 1.155 g.

^2)^ Nutrient levels were calculated values.

**Table S3** Primers sequences used for real time PCR

| **Genes** | **GenBank ID** | **Sequence (5′-3′)** | **Size (bp)** |
| --- | --- | --- | --- |
| *β-actin* | XM_021086047.1 | F: GGCACCACACCTTCTACAACGAG | 102 |
|  |  | R: TCATCTTCTCACGGTTGGCTTTGG |  |
| *ACC* | NM_001114269.1 | F: CAAAGAGGTTCCAGGCACAGTCC | 146 |
|  |  | R: CGTCAGCATGTCAGAAGGCAGAG |  |
| *ATGL* | NM_001098605.1 | F: TCACCAACACCAGCATCCA | 95 |
|  |  | R: GCACATCTCTCGAAGCACCA |  |
| *CPT-1* | NM_001129805.1 | F: TCAAAAACGGCAAGATGGGC | 155 |
|  |  | R: TGGAATGTTGGGGTTGGTGT |  |
| *FABP4* | NM_001002817.1 | F: CAGGAAAGTCAAGAGCACCA | 227 |
|  |  | R: TCGGGACAATACATCCAACA |  |
| *FASN* | NM_001099930.1 | F: TACCTTGTGGATCACTGCATAGA | 113 |
|  |  | R: GGCGTCTCCTCCAAGTTCTG |  |
| *HSL* | NM_214315.3 | F: GCGTGCTCTCCAAGTGTGTCAG | 135 |
|  |  | R: CCAGGCGGAGGTCTCGGAAG |  |
| *LPL* | NM_214286.1 | F: ACACAGTTGAGGACACTTGCCATC | 116 |
|  |  | R: TCCTGTCACCGTCCAGCCATG |  |
| *PPARα* | NM_001044526.1 | F: GGCACTGAACATCGAATGTAGAAT | 80 |
|  |  | R: TGCAACCTTCACAGGCATGA |  |
| *PPARγ* | NM_214379.1 | F: TCCATGCTGTCATGGGTGAA | 103 |
|  |  | R: ACCATGGTCACCTCTTGTGA |  |
| *SCD* | NM_213781.1 | F: ATTGGGAGCTGTGGGTGAG | 90 |
|  |  | R: AAGTTGATGTGCCAGCGGTA |  |
| *SREBP-1* | NM_214157.1 | F: GCGACGGTGCCTCTGGTAGT | 218 |
|  |  | R: CGCAAGACGGCGGATTTA |  |

*ACC*, acetyl-CoA carboxylase; *ATGL*, adipose triglyceride lipase; *CPT*-1, carnitine palmityl transferase-1; *FABP4*, fatty acid binding protein 4; *FASN*, fatty acid synthase; *HSL*, hormone sensitive lipase; *LPL*, lipoprotein lipase; *PPARα*, peroxisome proliferator-activated receptor α; *PPARγ*, peroxisome proliferator-activated receptor γ; *SCD*, stearyl coenzyme A desaturase; *SREBP-1*, sterol-regulatory element binding protein-1.

**Table S4** The richness and diversity of colonic microbiota in offspring piglets

| **Item** | **C group** | **SOA group** | **SOP group** | **SOS group** | **SEM** | ***P*-values** |
| --- | --- | --- | --- | --- | --- | --- |
| Chao1 | | | | | | |
| 65 day-old | 3836.91 | 3016.75 | 2869.47 | 3442.10 | 262.454 | 0.069 |
| 95 day-old | 3870.25 | 2974.35 | 2807.58 | 3437.23 | 294.549 | 0.085 |
| 125 day-old | 3268.84 | 3491.35 | 3179.43 | 3152.65 | 287.534 | 0.896 |
| Shannon | | | | | | |
| 65 day-old | 7.28 | 7.10 | 6.53 | 6.97 | 0.284 | 0.549 |
| 95 day-old | 7.44 | 7.43 | 7.18 | 7.35 | 0.350 | 0.927 |
| 125 day-old | 7.91 | 7.49 | 7.68 | 7.65 | 0.292 | 0.643 |
| Simpson | | | | | | |
| 65 day-old | 0.94 | 0.94 | 0.92 | 0.94 | 0.013 | 0.916 |
| 95 day-old | 0.95 | 0.94 | 0.93 | 0.94 | 0.016 | 0.831 |
| 125 day-old | 0.97 | 0.95 | 0.96 | 0.95 | 0.009 | 0.322 |
| Pielou’s | | | | | | |
| 65 day-old | 0.62 | 0.63 | 0.59 | 0.61 | 0.021 | 0.746 |
| 95 day-old | 0.65 | 0.66 | 0.65 | 0.65 | 0.026 | 0.965 |
| 125 day-old | 0.70 | 0.66 | 0.68 | 0.67 | 0.021 | 0.390 |

Data are expressed as means with the standard error of the mean (SEM). C, control group; SOA, antibiotic supplementation in sow-offspring diets; SOP, probiotics supplementation in sow-offspring diets; SOS, synbiotics supplementation in sow-offspring diets. The replicates per group at 65 day-old were 8. The replicates of the C, SOA, SOP, and SOS groups at 95 day-old were 8, 8, 8, and 7, respectively. The replicates of the C, SOA, SOP, and SOS groups at 125 day-old were 8, 5, 6, and 6, respectively.
